# Thymopoiesis in Pre- and Post-Hematopoietic Stem Cell Transplantation

**DOI:** 10.3389/fimmu.2018.01889

**Published:** 2018-09-07

**Authors:** Luis Klaus A. da Rocha, Samar Freschi de Barros, Francine Bandeira, Alexia Bollini, Lucia Helena de A. Testa, Anderson João Simione, Marina de O. e Souza, Lilian P. Zanetti, Leila Cibele S. de Oliveira, Ana Claúdia F. dos Santos, Mair Pedro de Souza, Vergílio Antônio R. Colturado, Jorge Kalil, Clarisse M. Machado, Luiza Guilherme

**Affiliations:** ^1^Laboratory of Immunology, Heart Institute (InCor), Clinical Hospital, University of São Paulo, São Paulo, Brazil; ^2^Hematopoietic Stem Cell Transplantation Sector, Amaral Carvalho Hospital, Jaú, Brazil; ^3^Laboratory of Virology, Tropical Medicine Institute (IMT), University of São Paulo, São Paulo, Brazil

**Keywords:** T-cell receptor excision circles, T-cell receptor gene, autologous hematopoietic stem cell transplantation, allogeneic hematopoietic stem cell transplantation, malignant hematological diseases, immune reconstitution, thymopoiesis, adaptive immune system

## Abstract

Hematopoietic stem cell transplantation (HSCT) is an important therapeutic option for some hematological diseases. However, patients who undergo HSCT acquire a state of immunodeficiency that causes significant mortality. Reconstitution of thymic function is needed to support the immune system. One way to measure thymic function is through T-cell receptor excision circle (TREC) quantification. TRECs are generated by T-cell receptor gene rearrangements during T-cell maturation in the thymus and represent a reliable marker for thymic output. In this study, we aimed to assess aging and malignant hematological diseases as two important factors that may influence thymic output before HSCT. We observed that patients before HSCT presented signal joint TREC (sjTREC) numbers lower than 606.55 copies/μg DNA (low values) compared with healthy individuals, with an odds ratio (OR) of 12.88 [95% confidence interval (CI): 5.26–31.53; *p* < 0.001]. Our results showed that a group of older individuals (≥50 years old), comprising both healthy individuals and patients, had an OR of 10.07 (95% CI: 2.80–36.20) for low sjTREC values compared with younger individuals (≤24 years old; *p* < 0.001). Multiple logistic regression analysis confirmed that both older age (≥50 years old) and malignant hematological diseases and their treatments were important and independent risk factors related to thymic function impairment (*p* < 0.001). The median sjTREC value for patients of all ages was significantly lower than the sjTREC median for the subgroup of older healthy individuals (≥50 years old; *p* < 0.001). These data suggested that patients before HSCT and healthy individuals exhibited age-dependent thymic impairment, and that prior treatment for hematological diseases may exacerbate aging-related deterioration of natural thymic function. Furthermore, we analyzed these patients 9 months post-HSCT and compared patients who underwent autologous HSCT with those who underwent allogeneic HSCT. Both groups of patients achieved sjTREC copy numbers similar to those of healthy individuals. We did not find a close relationship between impaired thymic function prior to HSCT and worse thymic recovery after HSCT.

## Introduction

The thymus, which produces T-lymphocytes, is known to shrink with aging; thus, many researchers have tried to elucidate the relationship between age and immune system dysfunction by examining losses and changes in circulating T-lymphocytes (1, 2). Nonetheless, decreased thymic output with aging may not affect peripheral T-lymphocytes, because there is a strong homeostatic compensatory mechanism that stimulates independent expansion of mature T-cells from the thymus. However, this expansion will result in a limited T-cell receptor (TCR) repertoire with a decreased ability to adapt to new antigens and infectious agents (3).

During maturation of T-lymphocytes in the thymus, four TCR genes (α, β, γ, and δ) encode two types of disulfide-linked heterodimers. The majority of TCRs expressed by peripheral T-lymphocytes are composed of α and β chains; however, an alternative TCR comprising γ and δ chains is also expressed by 2–10% of cells (4, 5). T-cell receptor excision circles (TRECs) are stable circular DNA episomes generated during excisional rearrangement of TCR genes during T-lymphocyte division and maturation in the thymus. This process is identical in approximately 70% of αβ-T-cells. As the rate of intracellular degradation of TREC is low and TRECs do not replicate, they remain constant in number, despite being diluted during T-lymphocyte proliferation. Accordingly, these molecules have been used as markers of recent thymic emigrants (5, 6).

Many clinical situations associated with aging have been described as possible causes of impaired thymic function, including changes in growth factor and cytokine expression, low hormone production, and bone marrow loss (7, 8). TREC quantification has been shown in various studies to be more reliable for evaluation of thymic function (9–16). A series of observations on TREC dynamics during human immunodeficiency virus (HIV) infection and treatment and in autologous and allogeneic hematopoietic stem cell transplantation (HSCT) confirmed the usefulness of TREC analysis as a quantitative marker for thymic function. Among these studies, some have shown that aging is an important contributor to damaged thymic function through decreased TREC quantification (17–19). Other studies have found a relationship between decreased TREC quantification and malignant disease treatment, or increased TREC quantification after highly active antiretroviral therapy (HAART) or HSCT as a sign of thymic function recovery (10, 20–26).

Accordingly, in this study, we focused on age and thymic function before HSCT in patients who had undergone treatment for malignant hematological diseases and observed their situation after HSCT.

## Materials and Methods

### Patients and Controls

This study involved patients diagnosed with a hematological malignancy. All patients had an Eastern Cooperative Oncology Group Performance Clinical Status Scale score of 0–1. The patients had not undergone any treatment for infectious diseases at the time of blood collection.

The patients were admitted to the HSCT Program at Amaral Carvalho Hospital, Jaú, São Paulo, between March 2015 and October 2016 (*n* = 111). No history of surgical thymectomy, splenectomy, or positive HIV diagnosis was reported. Among these patients, 39 underwent autologous HSCT (auto-HSCT) and 72 underwent allogeneic HSCT (allo-HSCT). A comparison with a group of healthy blood donors (control group) was performed (*n* = 54; Table 1).

**Table 1:** Characteristics of patients and healthy individuals.

	Patients	Healthy individuals	*p*
Cohort group, *n*	111 (67.3%)	54 (32.7%)	<0.001
Age (range), years	41 (18–60)	40 (19–60)	0.542
Sex (male/female)	65 (58.6%)/46 (41.4%)	27 (16.4%)/27 (16.4%)	0.301

Previous diagnosis			
Allo-HSCT	ALL, 30 (27.1%)	Absent	
	AML, 38 (34.2%)	Absent	
	MDS, 4 (3.6%)	Absent	
Auto-HSCT	HD, 7 (6.3%)	Absent	
	NHL, 9 (8.1%)	Absent	
	MM, 23 (20.7%)	Absent	

*ALL, acute lymphocytic leukemia; allo-HSCT, allogeneic HSCT; AML, acute myeloid leukemia; auto-HSCT, autologous HSCT; HD, Hodgkin’s disease; HSCT, hematopoietic stem cell transplantation; MDS, myelodysplastic syndrome; MM, multiple myeloma; NHL, non-Hodgkin’s lymphoma*.

The patients’ ages ranged from 18 to 60 years (mean 41 years) and healthy individuals’ ages ranged from 19 to 60 years (mean 40 years). The participants were classified into four age groups: 18–24, 25–35, 36–49, and 50–60 years old. Participants were also classified per previous disease diagnosis as follows: acute lymphocytic leukemia (ALL); myeloid diseases for acute myeloid leukemia (AML) and myelodysplastic syndrome (MDS); lymphomas for non-Hodgkin’s lymphoma (NHL) and Hodgkin’s disease (HD); and multiple myeloma (MM).

DNA samples were collected 15–30 days before HSCT, during appointments for clinical evaluation. Volunteer donors’ samples were collected from the Blood Transfusion Service for comparison. Venous blood (6 mL) was collected for leukocyte count determination and DNA extraction. The DNA samples were stored at −20°C until use. At the time of assessment, 14.4% of patients had acute leukemias with minimal residual disease, as detected by flow cytometry (AML ≥ 1% and ALL ≥ 0.1% of malignant cells), and 15.3% of patients had MM with partial response (PR) or very good PR, or lymphomas with PR without malignant medullar infiltration. Only three patients (2.7%) had received both chemotherapy and radiotherapy before HSCT. All patients had received their last disease treatment more than 1 month before blood collection, but only 17.1% had received their last treatment more than 6 months previously.

We examined the thymic recovery of these patients and collected new blood samples around the ninth month (270 ± 15 days) post-HSCT. During this period, 6 patients who underwent allo-HSCT died, and another 24 patients did not provide blood samples because they did not attend an appointment during the predefined time around the ninth month. A total of 81 patients were analyzed in this period (30 patients underwent auto-HSCT and 51 underwent allo-HSCT). We noted all the infections that needed hospital treatment or events related to acute and chronic graft-versus-host disease (GvHD) after HSCT. All patients undergoing allo-HSCT were human leukocyte antigen identical matched to donor.

### Signal Joint TREC (sjTREC) Quantification

Genomic DNA was extracted from blood samples using a QIAamp DNA Blood Minikit (27). Quantification of sjTRECs was performed by real-time quantitative polymerase chain reaction (qPCR) according to the MyTRECKit protocol (28).

Briefly, the kit was formulated with TaqMan dual-labeled fluorescent probes and a gene expression master mix containing antibody-mediated hot-start DNA polymerase, enhancers, stabilizers, dNTPs, MgCl^2^ cofactor, and a separate ROX reference dye. The reaction was set up with a final volume of 20 µL containing 16 µL of total mix (10 µL of gene expression master mix, 1 µL of primers and probes, 1 µL of reaction enhancer, and 4 µL of nuclease-free water) plus 4 µL DNA sample or 4 µL negative or positive control. The qPCR cycling program was as follows: one cycle at 95°C for 3 min for polymerase activation, followed by 40 cycles at 95°C for 30 s (denaturation) and 60°C for 1 min (annealing/extension) for amplification.

A standard curve was generated using the qPCR instrument (Applied Biosystems QuantStudio 12K Flex; Applied Biosystems, Foster City, CA, USA) by absolute quantification. FAM dye was used as a reporter for sjTREC, and VIC reporter was used for β-actin, an endogenous control. NFQ-MGB was used as a quencher for both genes. ROX dye was applied as a passive reference. The calibration curves were prepared using six different amplification points for both genes as follows: 10^6^, 10^5^, 10^4^, 10^3^, 10^2^, and 25 for the sjTREC standard curve; and 10^7^, 10^6^, 10^5^, 10^4^, 10^3^, and 10^2^ for the β-actin standard curve. Calibrator reactions for standard curves were set up in triplicate, and those for samples and controls were performed in duplicate.

The baseline was kept at cycle 3, and the threshold was fixed at 0.043745 for sjTREC and 0.09886 for β-actin. The slopes were between −3.58 and −3.10, with amplification efficiency values around 96–100% and a correlation coefficient (*R*^2^) greater than 0.99.

Signal joint TREC data were expressed as the number of sjTREC copies per μg of DNA after correlation with the endogenous control gene β-actin.

### Statistical Analysis

We used the Student’s *t*-test to compare ages of patients and healthy individuals. The sex frequencies were verified through chi-square tests (29). Previous disease diagnoses were also described in terms of absolute and relative frequencies.

Lymphocyte and sjTREC distribution values were evaluated for both the healthy individuals and the patients groups. sjTREC values were stratified by age group and previous disease diagnosis. Mann–Whitney tests were used for comparisons between two categories, and Kruskal–Wallis tests were used for multiple comparisons, followed by Dunn tests. To observe differences among age groups, we applied ANOVA with Bonferroni correction (29, 30).

Correlations between continuous variables (age and sjTREC copy number) were assessed using Spearman’s rank-order correlation.

All sjTREC results were classified as higher or lower values based on the median sjTREC copy number from healthy individuals (606.55 copies). The association between low sjTREC values and the three predefined variables (sex, age, and cohort group) were analyzed by univariate and multivariate logistic regression analyses (30).

The sjTREC copy numbers were also classified as higher or lower based on the median sjTREC copy number for the post-HSCT patients (166.36 copies). The frequencies among the variables were verified by chi-square tests, Fisher’s tests, or the likelihood ratio (29).

A generalized estimating equation was used to estimate the parameters of a generalized linear model to find possible correlations between the outcomes of the patients before and 9 months after HSCT (30).

The significance level was set to 0.05 for all tests performed. All *p*-values were based on two-sided tests, and all calculations were performed using IBM-SPSS version 20.0 and the MedCalc statistical software version 17.4 (2017).

## Results

We enrolled 165 individuals for the present study. They were divided in two groups: healthy individuals (*n* = 54) and patients (*n* = 111). All patients had a previous diagnosis of hematological malignant disease. The two groups were matched by age and sex (Table 1).

**Figure 1:**
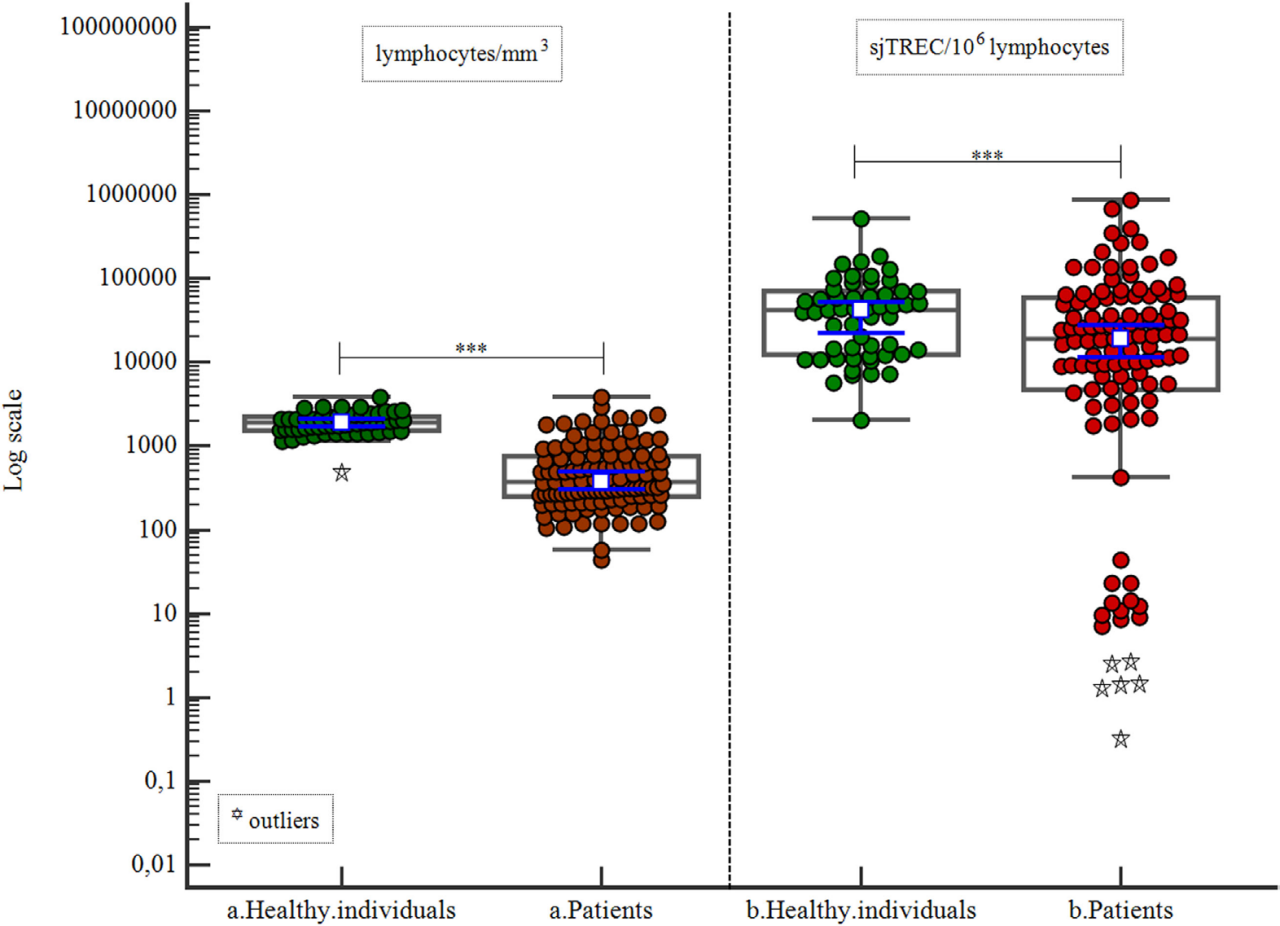
Correlation of lymphocyte count and signal joint TREC (sjTREC) count between healthy individuals and patients.

Healthy individuals were compared with patients in relation to lymphocyte count and sjTREC count by lymphocytes (10^6^). We found significant differences between the groups (*p* < 0.001 for lymphocyte count and *p* = 0.004 for sjTREC count; Table 2; Figure 1).

**Table 2:** Lymphocyte and signal joint TREC (sjTREC) counts.

Individuals	*n*	Variables	Minimum	Maximum	Median	25–75 percentile	*p*
a. Healthy individuals	54	Lymphocytes/mm^3^	493	3,822	1,897.5	1,548.0–2,240.0	
a. Patients	111	Lymphocytes mm^3^	44	3,88	378	252.25–757.06	<0.001
b. Healthy individuals	54	sjTREC/10^6^ lymphocytes	2,009.9	521,067.3	42,495.4	12,377.93–69,438.76	
b. Patients	111	sjTREC/10^6^ lymphocytes	0.32	873,700.28	19,099.71	4,781.55–57,693.64	0.004

**Figure 2:**
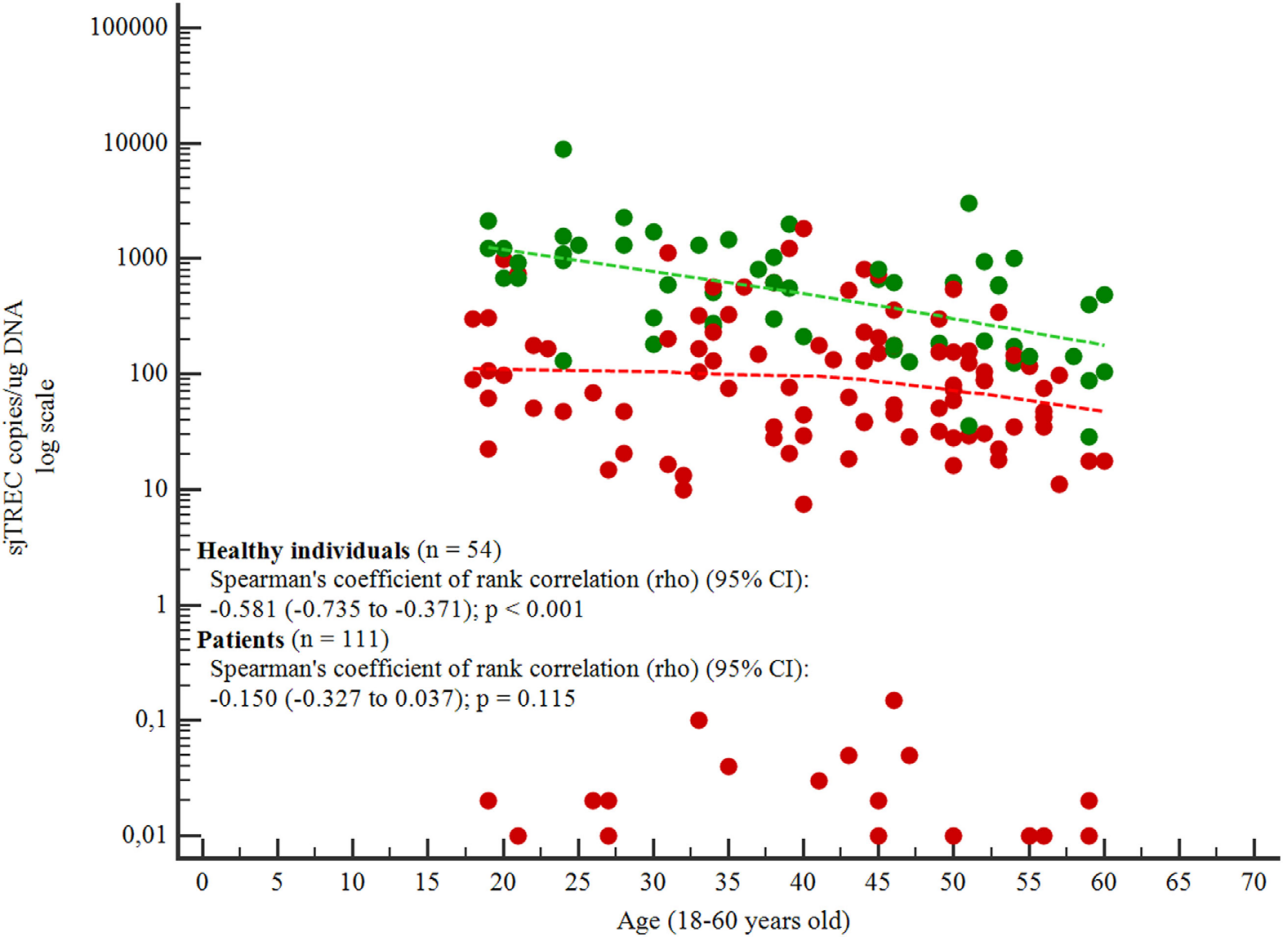
Correlation between age and signal joint TREC (sjTREC) count.

The ages of the individuals varied from 18 to 60 years old. With increasing age, the number of sjTREC copies/μg DNA decreased for both groups, but the decrease was significant only for the healthy individuals group (*p* < 0.001; Figure 2).

**Figure 3:**
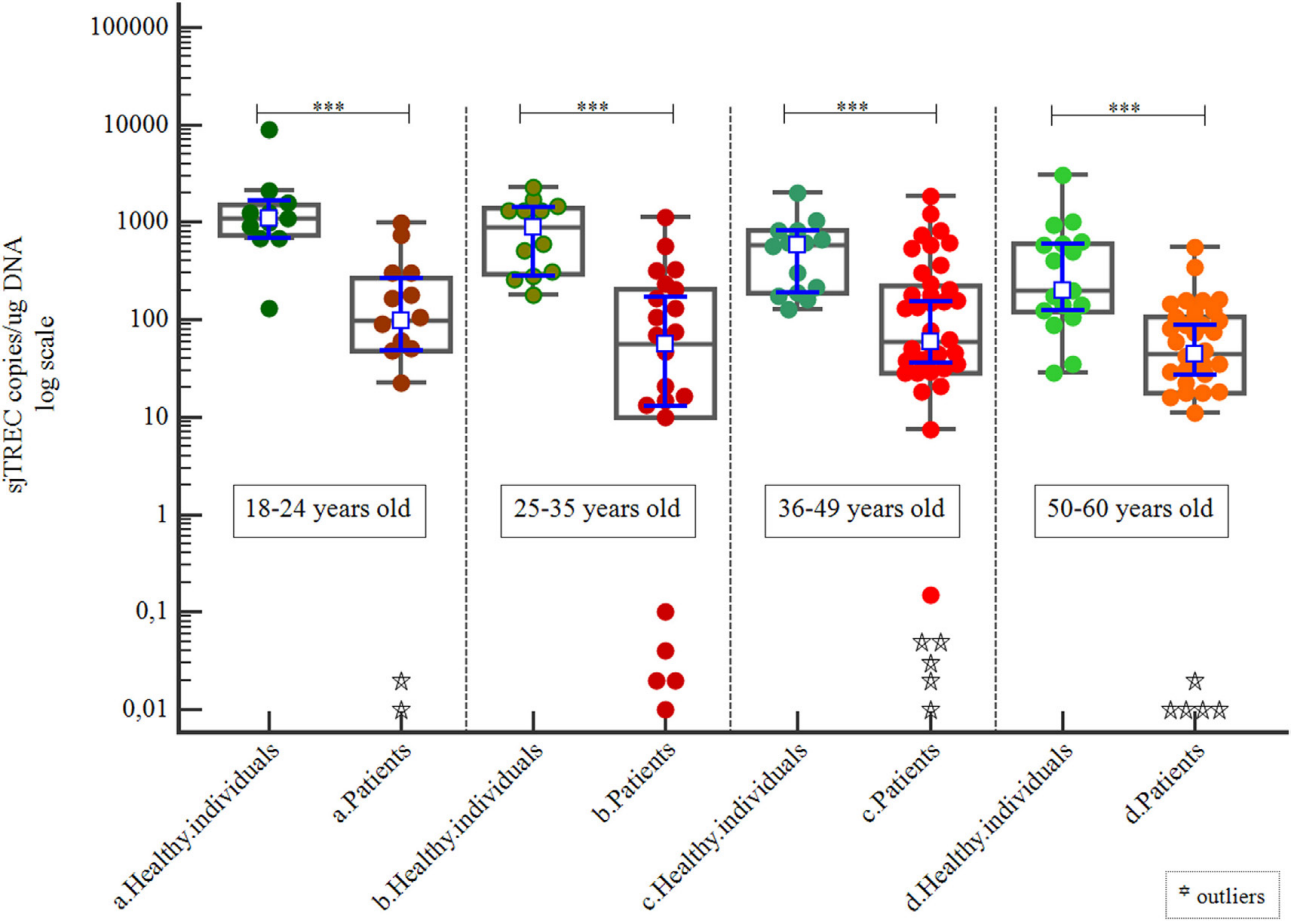
Correlation of signal joint TREC (sjTREC) count between healthy individuals and patients by age group.

When the cohort was separated by age group, we found that healthy individuals always had higher sjTREC copy numbers than patients (*p* < 0.001; Table 3; Figure 3).

**Table 3:** Signal joint TREC copies/μg DNA (according to Figures 3–5).

Figures	Individuals	*n*	Minimum	Maximum	Median	25–75 percentile	*p*
3	a. Healthy individuals (8–24 years old)	11	130.77	8,837.3	1,101.0	732.37–1,482.49	
	a. Patients (8–24 years old)	15	0.01	988.11	98.7	48.236–266.35	<0.001
	b. Healthy individuals (25–35 years old)	12	179.81	2,257.75	951.10	292.3–1,388.44	
	b. Patients (25–35 years old)	22	0.01	1,137.55	56.97	10–203.1	<0.001
	c. Healthy individuals (35–49 years old)	14	127.54	1,996.05	589.021	187.25–813.57	
	c. Patients (35–49 years old)	40	0.01	1,843.38	58.62	28.40–218.58	<0.001
	d. Healthy individuals (50–60 years old)	17	28.46	3,015.51	196.05	119.76–604.89	
	d. Patients (50–60 years old)	34	0.01	548.33	44.98	17.62–105.35	<0.001

4	Patients	111	0.01	1,843.38	61.59	18.21–163.74	
	Healthy individuals	54	28.46	8,837.3	606,48	187.25–1,101.0	
	Healthy individuals (50–60 years old)	17	28.46	3,015.51	196.06	119.76–04.89	<0.001*

5	a. Healthy individuals	54	28.46	8,837.3	606.48	187.25–1,101.0	
	b. Myeloid diseases	42	0.01	1,843.38	130.5	28.14–303.89	
	c. ALL	30	0.01	988.11	68.04	13.18–165.06	
	d. Lymphomas	16	0.01	729.66	61.47	19.84–111.59	
	e. MM	23	0.01	548.33	31.72	18.67–58.62	**

*The differences were significant between patients and healthy individuals (*p* < 0.001) and between patients and healthy individuals ≥50 years old (*p* < 0.001).

**The differences were significant between healthy individuals and patients distinguished by their previous hematological diseases (*p* < 0.001) and also between myeloid diseases and MM subgroups (*p* = 0.002).

**Figure 4:**
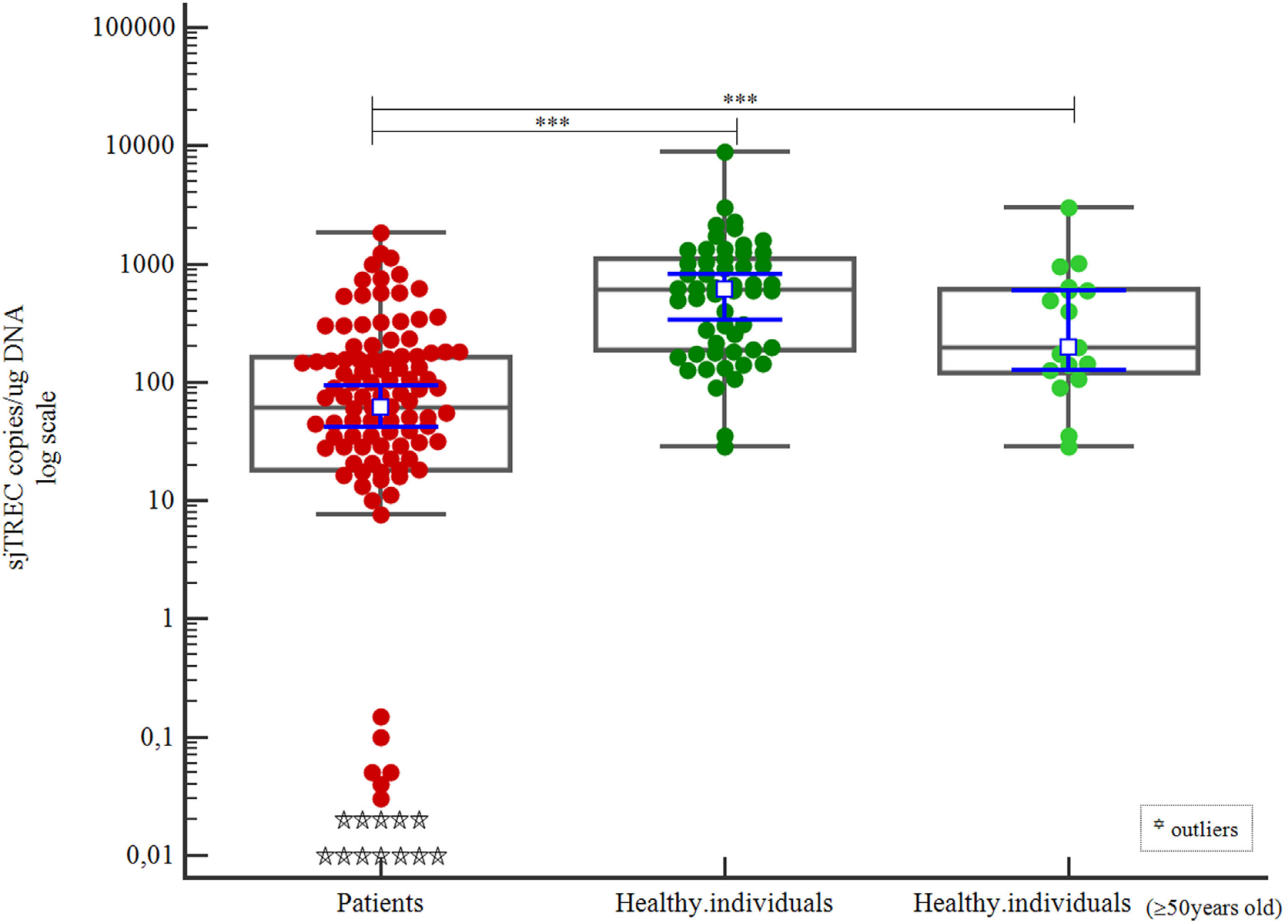
Correlation of signal joint TREC (sjTREC) count between patients and healthy individuals.

Patients had significantly lower sjTREC counts than healthy individuals, even in the case of the oldest subgroup of healthy individuals (*p* < 0.001; Table 3; Figure 4).

**Figure 5:**
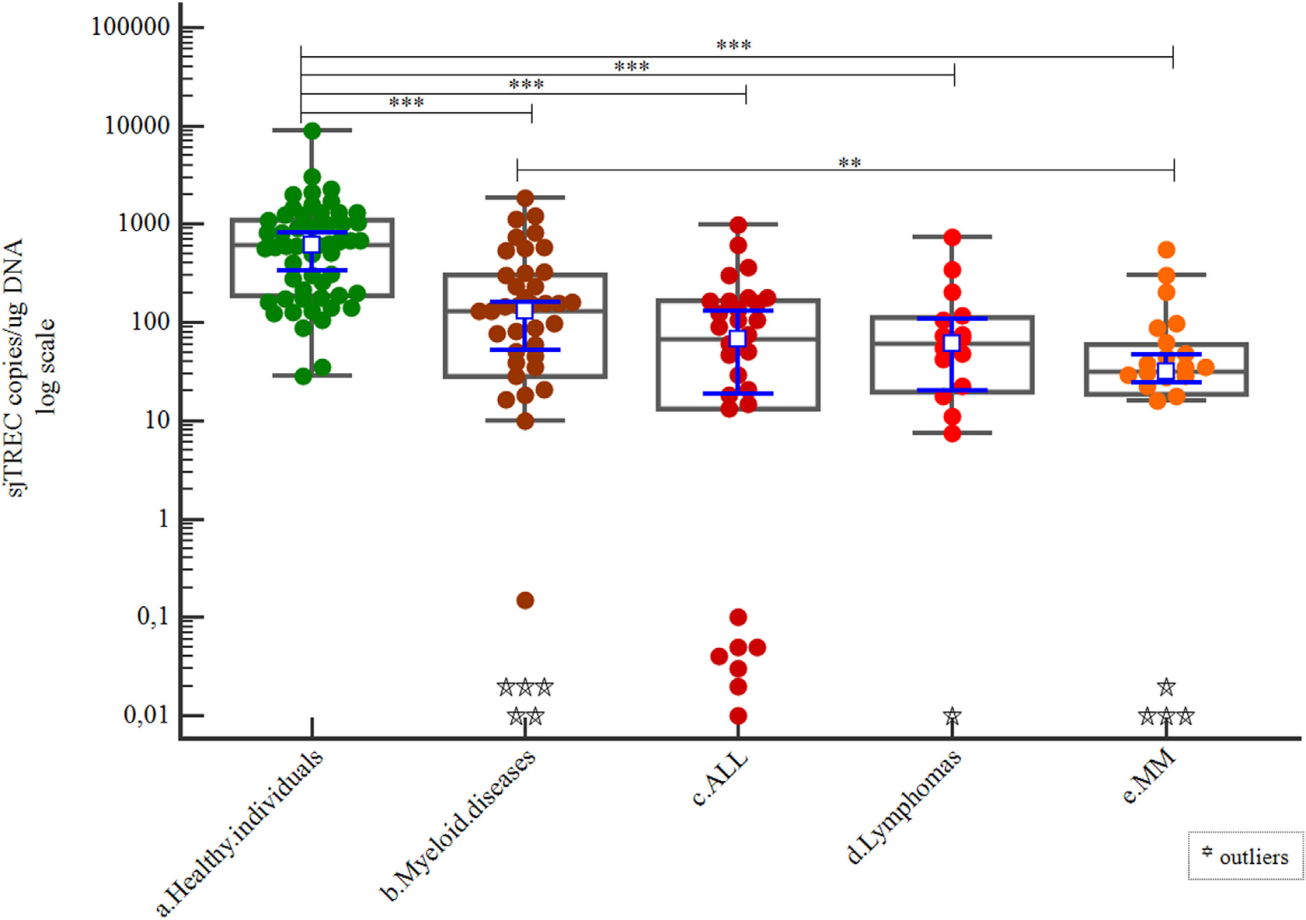
Correlations of signal joint TREC (sjTREC) count among healthy individuals and patient disease subgroups.

Patients were grouped per their previous diagnoses as described in the previous section. Healthy individuals had a greater number of sjTREC copies than any of these patient subgroups (*p* < 0.001). The myeloid diseases subgroup showed a greater number of sjTREC copies than the MM subgroup (*p* = 0.002; Table 3; Figure 5).

**Figure 6:**
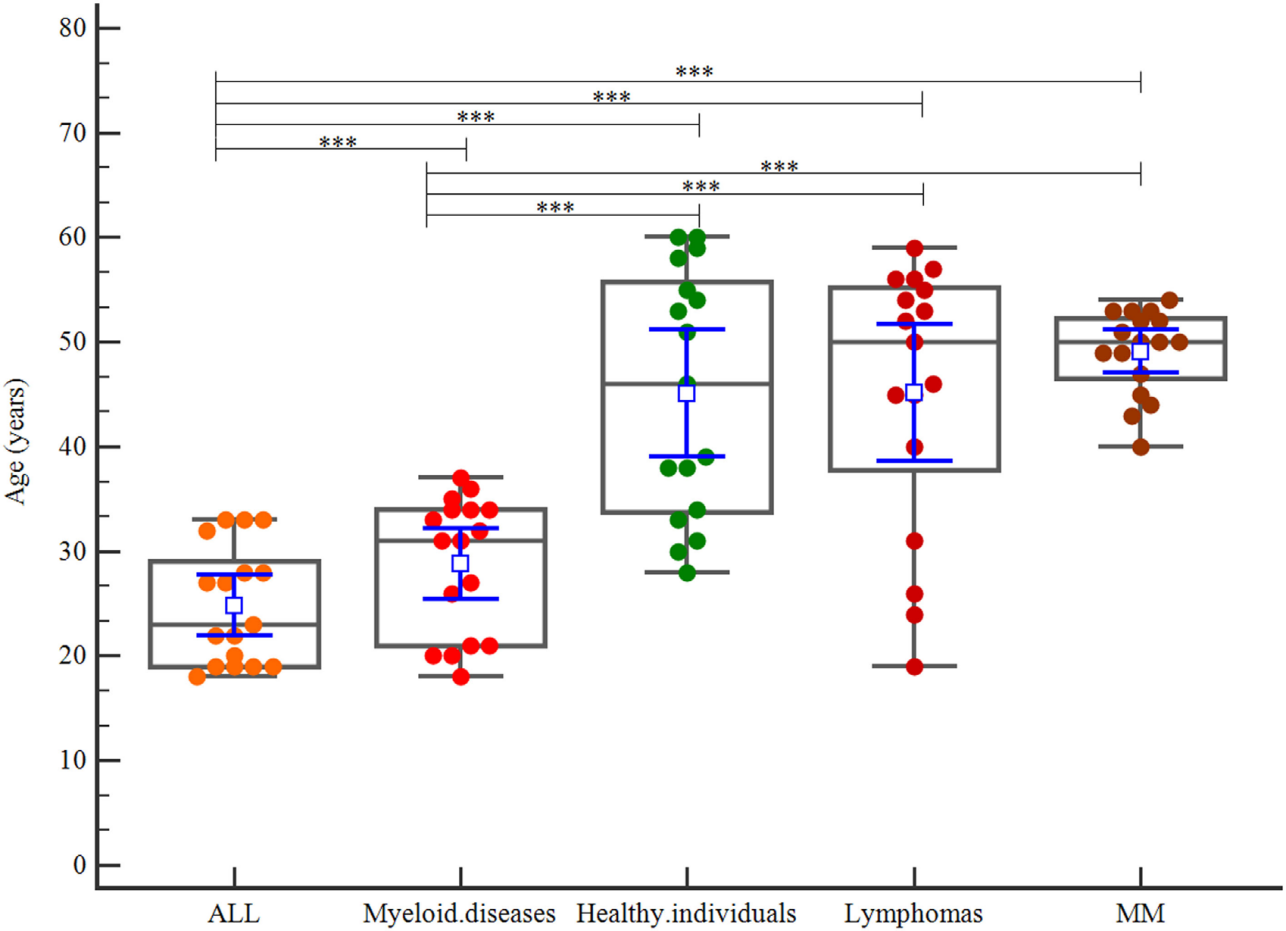
Correlation of age among patient disease subgroups and healthy individuals.

Taking age into consideration, the ALL group was the youngest group (*p* < 0.001). The myeloid diseases subgroup was younger than the healthy individuals group, and the lymphoma and MM subgroups (*p* < 0.001). The mean ages for ALL, myeloid diseases, healthy individuals, lymphomas, and MM were 24, 28, 45, 45, and 49 years, respectively (Figure 6).

The median number of sjTREC copies for healthy individuals was 606.55 copies; this value was used to set high and low values for subsequent analyses.

Univariate logistic regression analysis revealed higher odds ratios (ORs) for the three older age subgroups compared with the youngest subgroup (≤24 years old): OR = 3.31 for 25–35 years old (*p* = 0.039), OR 3.00 for 36–49 years old (*p* = 0.032), and OR = 10.07 for 50–60 years old (*p* < 0.001). Interestingly, the oldest subgroup had an OR that was three times higher for low values of sjTREC than those of the other subgroups.

Multiple logistic regression was applied to all populations studied (*n* = 165). Among the predefined groups, age was an important risk factor; for example, individuals at least 50 years old had an OR of 17.35 [95% confidence interval (CI) = 3.75–80.21, *p* < 0.001] for low values of sjTREC copies. Being in the patient group was considered another independent and important risk factor for low sjTREC copy number [OR = 17.25 (95% CI = 6.28–47.38), *p* < 0.001; Table 4].

**Table 4:** Univariate and multivariate logistic regression analyses with odds ratios (ORs) for low signal joint TREC (sjTREC) copy numbers (lower than the mean sjTREC copy number of 606.55) based on sex, age, and health condition (*n* = 165).

	sjTREC value									
Variable	Higher (*n* = 35)	Lower (*n* = 130)	OR not adjusted	95% CI		*p*	OR adjusted	95% CI		*p*
				Lower	Upper			Lower	Upper	
**Sex**										
Female	18 (24.7)	55 (75.3)	1.00				1.00			
Male	17 (18.5)	75 (81.5)	1.44	0.68	3.05	0.336	1.27	0.50	3.25	0.619
**Age, years**										
18–24	12 (46.2)	14 (53.8)	1.00				1.00			
25–35	7 (20.6)	27 (79.4)	3.31	1.06	10.27	0.039	4.67	1.13	19.27	0.033
36–49	12 (22.2)	42 (77.8)	3.00	1.10	8.18	0.032	2.84	0.80	10.03	0.105
50–60	4 (7.8)	47 (92.2)	10.07	2.80	36.20	<0.001	17.35	3.75	80.21	<0.001
**Cohort**										
Healthy individuals	27 (50)	27 (50)	1.00				1.00			
Patients	8 (7.2)	103 (92.8)	12.88	5.26	31.53	<0.001	17.25	6.28	47.38	<0.001

**Figure 7:**
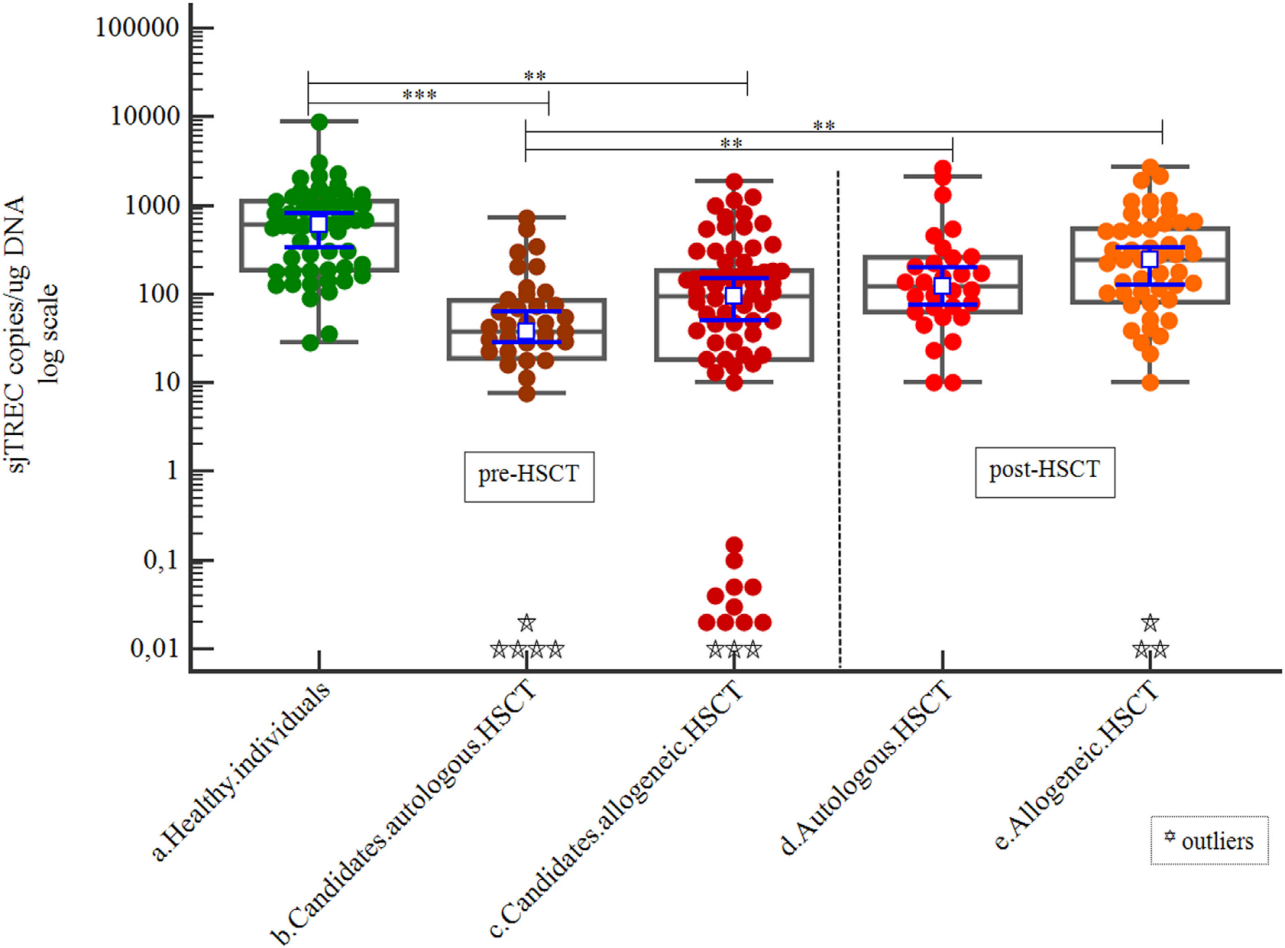
Correlation of signal joint TREC (sjTREC) count between healthy individuals and patients (as candidates for HSCT and post-HSCT).

Patients were analyzed at two time points relative to HSCT: as candidates for HSCT and 9 months post-HSCT. We also compared patients who underwent auto-HSCT with those who underwent allo-HSCT. There were no differences between the candidates for allo-HSCT and those for auto-HSCT, but both sets had significantly lower sjTREC copy numbers than healthy individuals. Both groups of patients showed good thymic recovery 9 months post-HSCT, i.e., both achieved sjTREC copy numbers similar to those of healthy individuals (Table 5; Figure 7).

**Table 5:** Correlation between types of HSCT (auto- and allo-) and time (pre-HSCT to the ninth month post-HSCT).

a. GEE with gamma distribution, identity binding function with first order autoregressive correlation matrix						

Variable	Wald test		df	*p*		
Type of HSCT	18.304		2	<0.001		
Time	15.911		1	<0.001		
Type of HSCT and time	0.007		1	0.932		
**b. Bonferroni multiple comparisons**						
					**95% CI**	
**Correlation between the variables**	**Mean**	**SE**	**df**	*p*	**Under**	**Upper**

Healthy individuals—pre-auto-HSCT patient	796.06	184.63	1	<0.001	277.79	1,314.33
Healthy individuals—post-auto-HSCT patient	538.32	206.54	1	0.092	−41.45	1,118.1
Healthy individuals—pre-allo-HSCT patient	681.96	186.94	1	0.003	157.21	1,206.71
Healthy individuals—post-allo-HSCT patient	435	206.03	1	0.347	−143.33	1,013.32
Pre-auto-HSCT patient—post-auto-HSCT patient	257.74	90.0	1	0.042	−510.37	−5.11
Pre-auto-HSCT patient—pre-allo-HSCT patient	114.1	43.29	1	0.084	−235.63	7.43
Pre-auto-HSCT patient—post-allo-HSCT patient	361.06	96.82	1	0.002	−632.85	−89.28
Post-auto-HSCT patient—pre-allo-HSCT patient	143.63	102.2	1	0.999	−143.25	430.52
Post-auto-HSCT patient—post-allo-HSCT patient	103.33	133.96	1	0.999	−479.36	272.71
Pre-allo-HSCT patient—post-allo-HSCT patient	246.96	88.93	1	0.055	−496.60	2.67

*allo-HSCT, allogeneic HSCT; auto-HSCT, autologous HSCT; df, degrees of freedom; GEE, generalized estimating equation; HSCT, hematopoietic stem cell transplantation*.

The median number of sjTREC copies from patients after HSCT was determined (166.36 copies), and we compared the characteristics listed in Tables 6 and 7 for both groups of HSCT (auto- and allo-HSCT) and correlated them with poor (lower sjTREC values) or good (higher sjTREC values) thymic recovery. Only episodes of cytomegalovirus (CMV) were related to poor thymic recovery in allo-HSCT patients (*p* = 0.030; Tables 6 and 7).

**Table 6:** Characteristics of patients 9 months post-autologous hematopoietic stem cell transplantation (HSCT).

Autologous-HSCT				

Variables		sjTREC/μg DNA (median: 166.36)			
		<median	≥median	*p*
			
		*n*(%)/*n*(%)/*n*(%)	*n*(%)/*n*(%)/*n*(%)	
Age groups	<50/≥50 years old	4 (22.2)/14 (77.8)	7 (58.3)/5 (41.7)	0.063
Sex	Male/female	3 (16.7)/15 (83.3)	6 (50)/6 (50)	0.102
Diagnosis	HD/NHL/MM	2 (11.1)/4 (22.2)/12 (66.7)	2 (16.7)/3 (25)/7 (58.3)	0.875
Treatment for a prior disease	Chemo/chemo plus radio	17 (94.4)/1 (5.6)	11 (91.7)/1 (8.3)	0.999
Bone marrow status before HSCT	CR/PR or VGPR	11 (61.1)/7 (38.9)	8 (66.7)/4 (33.3)	0.761
Time between last chemo/radio and HSCT	≥6 months/<6 months	1 (5.6)/17 (94.4)	4 (33.3)/8 (66.7)	0.158
Conditioning regimen (melphalan mg/m^2^)	200/<200	6 (33.3)/12 (66.7)	5 (41.7)/7 (58.3)	0.712
Stem cell source	PBSC	18 (100)	12 (100)	Not applied
Bacterial infection episodes	Not present/present	9 (50)/9 (50)	9 (75)/3 (25)	0.260
CMV infection episodes	Not present	18 (100)	12 (100)	Not applied
Fungal infection episodes	Not present/present	18 (100)/0	11 (91.7)/1 (8.3)	0.400
Clinical status	Alive	18 (100)	12 (100)	Not applied
Total	30 (100.0)	18 (100)	12 (100)	Not applied

*Chemo, chemotherapy; CMV, cytomegalovirus; CR, complete response; HD, Hodgkin’s disease; HSCT, hematological stem cell transplantation; MM, multiple myeloma; NHL, non-Hodgkin’s lymphoma; PBSC, peripheral blood stem cell; PR, partial response; radio, radiotherapy; sjTREC, signal joint TREC; VGPR, very good partial response*.

**Table 7:** Characteristics of patients 9 months post-allogeneic hematopoietic stem cell transplantation (HSCT).

Allogeneic-HSCT				

Variables		sjTREC/μg DNA (median: 166.36)			
		<median	≥median	*p*
			
		*n*(%)/*n*(%)/*n*(%)	*n*(%)/*n*(%)/*n*(%)	
Age groups	<50/≥50 years old	17 (77.3)/5 (22.7)	26 (89.7)/3 (10.3)	0.268
Sex	Male/female	11 (50)/11 (50)	12 (41.4)/17 (58.6)	0.540
Diagnosis	ALL/AML/MDS	9 (40.9)/12 (54.5)/1 (4.5)	12 (41.4)/15 (51.7)/2 (6.9)	0.932
Treatment for a prior disease	Chemo/chemo plus radio	21 (95.5)/1 (4.5)	28 (96.6)/1 (3.4)	0.999
Bone marrow status before HSCT	MRD/active^a^/CR	7 (31.8)/1 (4.5)/14 (63.6)	6 (20.7)/2 (6.9)/21 (72.4)	0.650
Time between last chemo/radio and HSCT	≥6 months/<6 months	4 (18.2)/18 (81.8)	5 (17.2)/24 (82.8)	0.999
Conditioning regimen	MAC/RIC	12 (54.5)/10 (45.5)	23 (79.3)/6 (20.7)	0.059
Stem cell source	PBSC/BMSC	10 (45.5)/12 (54.5)	11 (37.9)/18 (62.1)	0.589
Acute-GvHD	Absent/present	10 (45.5)/12 (54.5)	12 (41.4)/17 (58.6)	0.771
Chronic-GvHD	Absent/present	9 (40.9)/13 (59.1)	15 (51.7)/14 (48.3)	0.443
Bacterial infection episodes	Not present/present	7 (31.8)/15 (68.2)	8 (27.6)/21 (72.4)	0.743
CMV infection episodes	Not present/present	1 (4.5)/21 (95.5)	9 (31)/20 (69)	0.030
Fungal infection episodes	Not present/present	21 (99.5)/1 (4.5)	25 (86.2)/4 (13.8)	0.375
Clinical status	Dead/alive	4 (18.2)/18 (81.8)	2 (6.9)/27 (93.1)	0.383
Total	51 (100%)	22 (100)	29 (100)	Not applied

*ALL, acute lymphocytic leukemia; AML, acute myeloid leukemia; chemo, chemotherapy; BMSC, bone marrow stem cell; CMV, cytomegalovirus; CR, complete response; GvHD, graft-versus-host disease; HD, Hodgkin’s disease; HSCT, hematological stem cell transplantation; MAC, myeloablative conditioning; MDS, myelodysplastic syndrome; MRD, minimal residual disease; MM, multiple myeloma; PBSC, peripheral blood stem cell; PR, partial response; radio, radiotherapy; RIC, reduced intensity conditioning; sjTREC, signal joint TREC*.

*^a^Active: three cases of MDS have not received any treatment before HSCT*.

Finally, the patients 9 months post-HSCT who had sjTREC copy numbers greater than the median value observed for healthy individuals (≥606.55 sjTREC copies; *n* = 15) were more likely to suffer from infections, despite many of these patients being less than 50 years old and having shown complete medullary remission before HSCT. In addition, the majority of these patients underwent allo-HSCT and a myeloablative conditioning regimen (Table 8).

**Table 8:** Characteristics of patients after the ninth month post-HSCT with sjTREC copies/μg DNA higher than the median of sjTREC value observed for healthy individuals (605.55 sjTREC copies/μg DNA).

Patients with sjTREC ≥ 605.55 copies/μg DNA			

Variables		*n*(%)/*n*(%)/*n*(%)*n*(%)	*p*
Age groups	<50/≥50 years old	13 (86.7)/2 (13.3)	0.005
Sex	Male/female	10 (66.7)/5 (33.3)	0.197
Diagnosis	ALL/AML/NHL/MM	6 (40)/6 (40)/1 (6.7)/2 (13.3)	0.137
Treatment for a prior disease	Chemo/chemo plus radio	15 (100)/0	Not applied
Bone marrow status before HSCT	MRD/CR/PR or VGPR	2 (13.3)/11 (73.3)/2 (13.3)	0.005
Time between last chemo radio and HSCT	≥6 months/<6 months	4 (26.7)/11 (73.3)	0.071
Type of HSCT	Allo-/auto-	12 (80)/3 (20.0)	0.020
Conditioning regimen	MAC/RIC/melphalan 200/melphalan < 200	9 (60)/3 (20)/2 (13.3)/1 (6.7)	0.016
Stem cell source	PBSC/BMSC	6 (40)/9 (60)	0.439
Acute-GvHD	Allo-HSCT: absent/present/or auto-HSCT	4 (26.7)/8 (53.3)/or 3 (20)	0.247
Chronic-GvHD	Allo-HSCT: absent/present/or auto-HSCT	5 (33.3)/7 (46.7)/or 3 (20)	0.449
General infection episodes	Not present/present	2 (13.3)/13 (86.7)	0.005
Total	Subgroup	15 (100)	Not applied

*ALL, acute lymphocytic leukemia; allo-HSCT, allogeneic HSCT; auto-HSCT, autologous HSCT; AML, acute myeloid leukemia; chemo, chemotherapy; BMSC, bone marrow stem cell; CR, complete response; GvHD, graft-versus-host disease; HSCT, hematopoietic stem cell transplantation; MAC, myeloablative conditioning; MM, multiple myeloma; MRD, minimal residual disease; NHL, non-Hodgkin’s lymphoma; PBSC, peripheral blood stem cell; PR, partial response; radio, radiotherapy; RIC, reduced intensity conditioning; sjTREC, signal joint TREC; VGPR, very good partial response*.

## Discussion

Age-related changes in the immune system may contribute to morbidity and mortality, mainly owing to decreased resistance to infection.

Moreover, the persistence of thymopoiesis and generation of TCR diversity has important implications for immunosenescence. Thymic function gradually decreases after puberty, with progressive changes in the thymic stroma for adipose tissue. However, in recent years, the adult thymus has been shown to remain active late in life and can still generate functional T-cells for the peripheral lymphoid repertoire, even in older individuals (>60 years old) (3).

Poor thymic function after HSCT is associated with higher morbidity and mortality. Immediately after HSCT, the thymus shows a temporary decrease in activity, and the circulating post-transplant T-cells derived from both residual mature T-cells present in the donor graft and *de novo* T-cells of the recipient from transplanted donor stem cells provide primary protection against external agents. However, thymic recovery speed is essential for quicker and broader immune responses, and clinical aspects other than age may also affect thymic function after HSCT (10, 19, 23–26, 31). Thus, in this study, we examined the relationships among age, patient condition, and thymic function before HSCT and characterized thymic output at the time of HSCT and the patient features associated with the index of thymic recovery.

Some studies have shown that aging is related to a gradual reduction of thymic function, and an inverse relationship between TREC quantity and aging has been reported. Steffens and colleagues studied the impact of aging on thymic output in the first 5 years of life in 121 children, and compared the results with those from 60 healthy individuals aged 23–58 years old. They found inverse correlations between age and coding joint TREC (cjTREC) levels in both groups (32). Similar results were observed by Geenen and colleagues for sjTRECs in 41 individuals between 1 and 83 years of age (5). These data were further supported by the results of Naylor and colleagues, who studied sjTRECs in CD4 T-cells in a cohort of individuals ranging from 18 to 90 years of age (3).

Serana and colleagues evaluated sjTRECs from peripheral blood mononuclear cells (PBMCs) in 37 patients with common variable immunodeficiency and compared the results with those from 78 healthy blood donors. They found a decrease in the number sjTRECs over time, with women showing higher values than men (33). Moreover, in a group of 172 Korean volunteers aged 16–65 years old, Cho and colleagues found a negative correlation between sjTREC levels and age (14). Consistent with these findings, our results demonstrated a negative correlation between sjTREC levels and age, although this was more evident in healthy individuals than in patients.

Interestingly, we did not find any differences in median sjTREC numbers between patients who underwent allo-HSCT and those who underwent auto-HSCT. However, the median sjTREC number was higher in patients with myeloid diseases than in patients with MM, and lower in both these groups than in healthy individuals. Thus, malignant hematological diseases and/or prior treatment may have caused similar thymic dysfunction in all patient groups, independently of the type of disease or treatment. In particular, patients with MM showed the lowest median sjTREC and also the highest mean age. Thus, although all groups showed some thymic dysfunction, the dysfunction was more evident in patients with MM, likely owing to the age of individuals in this group.

Thymic atrophy is not only a consequence of aging. Variations in thymic size and thymocyte numbers have also been noted in various physiological or pathological states, including puberty, pregnancy, thymoma, inflammation, exercise, infections, stress, exposure to substances, GvHD, acute respiratory distress, and malnutrition (1). Many of these processes are transient and reversible, in contrast to thymic involution and aging.

According to Taub and Longo, T-cell recovery after any transitory acquired injury is most likely to be a reflection of thymus responsiveness to increased demand. Similar to the bone marrow, the thymus may respond to stress insults with transient thymic cellularity alterations, which often depend on the type of stimulus and the grade and time of exposure. Under certain circumstances, such as during periods of chronic stress or repeated stimuli, thymic size and function are restored and, in some cases, enhanced responses may be observed. A number of drugs have been shown to induce a transient and reversible involution (7).

Douek and colleagues studied the relationships between age and sjTRECs/cjTRECs in PBMCs from birth to 73 years of age in healthy individuals, and compared the findings with those from patients who had undergone total thymectomy and patients who were infected with HIV during the early stages of the disease. Notably, the TREC numbers were found to decrease with increasing age, both after thymectomy and during HIV infection, but showed improvement after the introduction of HAART (21).

Zhang and colleagues measured α1-circles (89-kb TRECs) in a cross-sectional cohort study of 532 healthy individuals ranging in age from infants to 95 years old. They compared 126 HIV-1-infected adults who had never received antiretroviral therapy with 88 age-matched seronegative controls, and compared 42 HIV-1-infected children (0–10 years of age) with 124 age-matched seronegative controls. Individuals infected with HIV in both groups had fewer α1-circles than controls. Moreover, they observed that the number of α1-circles in healthy individuals was relatively stable for the first 10–15 years, decreased sharply at around 20 years of age, and continued to decrease gradually thereafter (34).

Signal joint TRECs have also been studied in relation to acute cardiac allograft rejection. A negative correlation between sjTREC levels and age was observed in 66 healthy volunteers and in 27 heart transplant recipients. Cardiac transplant recipients showed lower sjTREC levels than age-matched healthy controls (35). In addition, sjTREC levels were evaluated in 149 samples from individuals who had adverse drug reactions (anaphylaxis, urticaria, eosinophilia, and systemic symptoms) and compared with those from a previous report of 172 healthy individuals. The mean sjTREC level for individuals with altered immunological status was significantly lower than that of healthy individuals. Thus, immunological status was a factor affecting the accuracy of age prediction using sjTREC quantification (18).

Some studies have reported decreased TREC numbers and poorer thymic function following treatment in patients with hematological diseases. For example, Svaldi and colleagues tested cjTRECs in 25 patients with MM whose samples were collected between the first and second HSCT. They found a correlation between cjTREC levels and aging; patients younger than 50 years old had higher levels of cjTRECs than older patients. Moreover, HSCT resulted in a marked decrease in cjTRECs, followed by recovery 12 months after HSCT (23). Li and colleagues studied sjTRECs in 88 untreated AML cases and compared them with 38 healthy individuals and with 10 AML cases in remission (AML-CR) as controls. The sjTREC copy numbers in the AML-CR group did not differ from those in the healthy individuals group; however, the sjTREC levels were lower in patients with AML than in healthy individuals, even after adjustment for age. They also found that sjTREC levels were lower in patients in the AML group who were ≥40 years or older, whereas no difference was observed in the same subgroups of healthy individuals (36).

Sun and colleagues analyzed sjTRECs in 134 patients with diagnoses of HD and NHL aged 18–67 years. Serial analyses of structural changes in thymus showed that hyperplasia occurred in 28.4% of patients within a median of 4 months after chemotherapy. Moreover, sjTREC levels approached a nadir at the end of treatment and rose significantly to reach pretreatment levels 6 months after the end of chemotherapy (37). In addition, Haining and colleagues studied T-cell homeostasis in 73 children with newly diagnosed ALL who had just started treatment. The median age of the patients in this study was 4 years old (range 1–17 years) and the sample exhibited a bimodal distribution, with a second age peak at 15 years old. This group was compared with a data set from 805 healthy children ranging from 0 to 18 years old. sjTREC levels were measured in CD3^+^ T-cells. At diagnosis, sjTREC levels in patients with ALL were significantly lower than in healthy children, and remained low with respect to age-matched healthy controls during treatment. After 2 years of treatment, 86% of sjTREC values were less than the 10th percentile in healthy controls. The median sjTREC level in children treated for ALL was equivalent to that of 49-year-old controls. These data suggested that thymopoiesis was markedly reduced both in newly diagnosed and chemotherapy-treated patients with ALL, and that it failed to recover at any point during the 2-year treatment course (38).

Our data showed that thymopoiesis decreased with aging in both healthy individuals and patients. However, sjTREC levels were significantly higher in healthy individuals than in patients, regardless of age. We also found that the median number of sjTREC copies from all patients was significantly lower than that of the oldest healthy individuals (≥50 years old). Overall, these data suggest that thymic function was reduced in patients because of their previous conditions, probably as a consequence of disease status and prior treatments.

Immediately after the HSCT, peripheral T-cell proliferation can be induced to quickly restore the homeostatic pool, because thymic output is limited and slow. We believed that aging and clinical condition prior HSCT resulting from malignant diseases and their treatments would affect thymic output after HSCT. However, independently of the type of HSCT, we could not determine whether some features were related to a worse or a better thymic recovery. The only exception was CMV infection episodes, which were linked to worse thymic recovery in patients who underwent allo-HSCT.

It was particularly notable that most of the patients who showed the best thymic recovery after 9 months post-HSCT were less than 50 years old and presented complete response for disease treatment prior to HSCT.

In the future, a better understanding of the causes of thymic senescence may lead to the identification of approaches to prevent, or at least delay, thymic dysfunction. Even though we have not found a close relationship between impaired thymic function prior to HSCT and worse thymic recovery after HSCT, it is plausible to imagine that patients may have better clinical conditions to face the procedure if they have better thymic function prior to HSCT.

## Ethics Statement

This study was carried out in accordance with the recommendations of CNS no. 466/12 Resolution - CEP, Brazil, name of committee: Comitê de Ética em Pesquisa - Medicina USP. The protocol was approved by the Profa. Dra. Maria Aparecida de Azevedo Koike Folgueiras. All subjects gave written informed consent in accordance with the Declaration of Helsinki.

## Author Contributions

LR obtained informed consent from all participants, was responsible for the collection and storage of clinical and laboratory data, extracted and quantified DNA from healthy individuals, tested all samples by qPCR, and was responsible for statistical analysis and manuscript writing. SB supported LR in performing agarose gel electrophoresis, DNA quantification, and qPCR. FB, AB, LZ, and LO extracted and quantified DNA samples from the patients and registered the data. LT supported LR in obtaining informed consent, organized patient lists, and supervised blood sample collection. ACFdS supported LR in obtaining informed consent and supervised blood sample collection. AJS supported LR in performing statistical analysis and clinical data collection. MdOS was responsible for DNA extraction technique and sample storage, and oversaw the activities of FB, AB, LZ, and LO. MPdS and VC were responsible for maintaining medical records, recruiting patients, and supporting clinical data collection. JK was responsible for all research activities at the Laboratory of Immunology and critically contributed to this research. CM was responsible for all research activities at Amaral Carvalho Hospital and supported LR in study design and conceptualization, and in preparation of the consent form. LG acted as the general supervisor for the study, supported LR in the study design and conceptualization, and assisted with writing and revising the manuscript.

## Conflict of Interest Statement

The authors declare that the research was conducted in the absence of any commercial or financial relationships that could be construed as a potential conflict of interest.
